# One-hole split endoscope technique for migrated lumbar disc herniation: a single-centre, retrospective study of a novel technique

**DOI:** 10.1186/s13018-023-03967-3

**Published:** 2023-07-05

**Authors:** Yuhong Zhang, Bo Feng, Huaxiu Ning, Guohua Dai, Weiliang Su, Huaiwang Lu, Peng Hu

**Affiliations:** 1 Department of Spine Surgery, Binzhou Medical University Hospital, No. 661, Huanghe 2th Road, Binzhou, 256603 Shandong China; 2 Department of Neurology, Binzhou Medical University Hospital, No. 661, Huanghe 2th Road, Binzhou, 256603 Shandong China

**Keywords:** Migrated lumbar disc herniation, One-hole split endoscope technique, Clinical efficacy, Complication

## Abstract

**Background:**

Lumbar disc herniation (LDH) is one of the most common diseases of the spine, and migrated LDH is a more serious type, associated with nerve root function injury or abnormality. Regarding the increasing surgery adoption of treating migrated LDH, we aimed to investigate the clinical efficacy and safety of discectomy with a novel technique–one-hole split endoscope (OSE) technique.

**Methods:**

This was a retrospective analysis of migrated LDH treated between December 2020 and September 2021. Hospitalization time, operative duration, intraoperative blood loss, number of fluoroscopy exposures, incision length, postoperative facet preservation rate, number of excellent–good cases, lower back and leg visual analogue score (VAS), Oswestry Disability Index (ODI) and surgical complications were compared between high-grade migration group (82 cases) and low-grade migration group (148 cases). The Macnab criteria was used to evaluate the clinical outcome. The Shapiro‒Wilk test was used to test measurement data, and the *χ*^*2*^ test was used to test counting data.

**Results:**

There was no significant difference in hospitalization time, operative duration, intraoperative blood loss, number of fluoroscopy exposures, incision length or postoperative facet preservation rate between the two groups by independent sample *t* test or nonparametric test. At any time point, the lower back and leg VAS and ODI of the two groups were significantly improved compared to those before the operation, but there was no significant difference between the two groups at the same time point by two-way repeated measures ANOVA. There were two cases of postoperative nerve root stimulation symptoms in the high-grade migration group and three cases in the low-grade migration group. There was one patient reoperated in the high-grade migration group. There was no significant difference in number of excellent–good cases between the two groups. The overall excellent–good rate was 89.6%.

**Conclusion:**

The OSE technique has the advantages of less trauma, faster recovery, complete removal of the nucleus pulposus and a satisfactory early clinical efficacy in the treatment of migrated LDH.

## Introduction

Lumbar disc herniation (LDH), as one of the most common clinical spinal diseases, has attracted widespread attention, of which the migrated type accounts for approximately 35–72% [1, 2]. For LDH patients who have failed conservative treatment, surgery is still a choice [3]. Lee et al. divided the prolapse site into four zones: the dissociation of the nucleus pulposus to zones two and three was called the low-grade migration type, and zones one and four were called the high-grade migration type [4]. In the past, as a classical surgical decompression for the treatment of different types of LDH, open discectomy yielded complete decompression, but it also resulted in high trauma, slow postoperative recovery and excessive damage to the posterior column structure of the spine, which led to chronic low back pain and increased the risk of accelerated degeneration of adjacent segments after surgery [5, 6]. Although percutaneous endoscopic lumbar discectomy (PELD) has become an alternative to LDH, it is still considered challenging to achieve complete nerve decompression for high-grade migrated LDH due to factors such as the coaxial axis of the working channel and observation channel, fixed visual field, limited bony anatomical structure of the lateral approach and small range of movement of the interlaminar approach [6, 7]. In recent years, the unilateral biportal endoscope (UBE) technique has been popular in minimally invasive spinal surgery due to its advantages of flexible operation, less trauma and fast recovery. The treatment of lumbar disc herniation, including the high-grade migration type, has a significant efficacy [8, 9].

The one-hole split endoscope (OSE) technique was first proposed and applied in the clinic in 2019 in China. Similar to the UBE technique, the OSE technique is divided into a working channel and an observation channel, but the two channels of this technique are located in the same soft incision, separated from each other, and each can rotate and swing freely without fixed channel restriction. There is no V-shaped angle compared with the UBE technique, and the working channel and the observation channel have a higher degree of synchronization. Our department has applied the OSE technique to treat a large number of patients with lumbar spine-related diseases. Our guiding hypothesis is that OSE technique, similar with UBE technique, will has satisfactory early clinical efficacy and safety in different degree of migrated LDH. Thus, the purpose of this retrospective study was to investigate the safety and efficacy of discectomy with the OSE technique in the treatment of migrated LDH.

## Materials and methods

### Inclusion and exclusion criteria

This study involving human participants was in accordance with the ethical standards of the 1964 Declaration of Helsinki and its later amendments. Ethics committee approval and due consent were also obtained.

We retrospectively analysed 230 patients who were diagnosed with migrated LDH between December 2020 and September 2021 in a single centre at our institution. The anterior and lateral position and dynamic position X-ray, CT and MRI examination of lumbar vertebrae were obtained from all patients. Patients were eligible for inclusion in this study if they (i) Lower back pain and radiating pain or numbness in the lower limbs; (ii) MRI examination showing that a single segment nucleus pulposus tissue was migrated, and the responsible segment was consistent with the symptoms and sign; and (iii) regular conservative treatment (medication, rehabilitation) for 12 weeks was not effective or led to worsened symptoms, which seriously affected work and life. The exclusion criteria were as follows: (i) Greater than Meyerding I° lumbar spondylolisthesis, or lumbar spine instability [10]; (ii) intervertebral disc diseases, such as spondylodiscitis, intervertebral space infection, tumours, tuberculosis; or (iii) serious cardiovascular and cerebrovascular diseases, coagulation disorders and other surgical contraindications.

To assess the efficacy of the OSE technique in different degrees of migrated LDH more precisely, patients were categorized into two groups based on LEE criteria [4]—high-grade migration group and low-grade migration group. All the surgeries were undertaken by the same senior surgeon.

### Surgical technique

#### Anaesthesia and position

After anaesthesia was induced successfully, patients were placed in the prone position on a radiolucent surgery table, with the abdomen suspended, and the lumbar bridge slightly flexed to slightly open the responsible intervertebral space.

#### Preoperative preparation

Conventional disinfection cloths were applied. The endoscope, RF electrode knife, grinding drill and perfusion system were connected. C‐arm X‐ray fluoroscopy was used to confirm the target segment.

#### Establishment of working channel

Taking the operation on the left as an example, a longitudinal incision approximately 1.5 cm long was made on the left side of the intersection between the horizontal line of the responsible intervertebral space and the spinous process. The skin, subcutaneous tissue and deep fascia were cut in turn. The dilator expanded the soft tissue step by step to the bony surface of the lamina for blunt separation. The OSE and operating instruments were inserted into the incision, and the perfusion system was opened. A total of 3000 ml of isotonic saline was selected as the flushing fluid and placed at a level of approximately 50–60 cm above the operation area.

#### Procession of the microscopic field of view

A low-temperature plasma radio frequency cutter head was used to handle the interlaminar soft tissue and the surface tissue of the ligamentum flavum, revealing the left superior lamina inferior edge, lower lamina margin, ligament flavum, root of the spinous process and medial edge of the superior and inferior articular processes. Centred on the free prolapsed nucleus pulposus, high-speed dynamic grinding drills and a laminar rongeur were used to remove the corresponding lamina bone to the attachment point of the ligament flavum and the adhesion between the dural sac and the ligament flavum. The left part of the ligament flavum was removed, and the dural sac, nerve roots and intervertebral disc were exposed.

#### Removal of the free nucleus pulposus

Under the endoscope, a nerve retractor was used to protect the dural sac and nerve root from injury, and a nerve exfoliation ion was used to peel off the adhesion and explore the free intervertebral discs and annulus fibrosus. A pituitary rongeur was used to remove protruding and loose nucleus pulposus tissue in the intervertebral space. By moving the working channel, migrated nucleus pulposus tissue in the spinal canal was removed, and scar tissue or calcified adhesion around the nerve root was properly excised to ensure full decompression. Finally, the annular break was wrinkled and shaped by the radio frequency.

#### Suture

Bipolar radio frequency was used for annulus fissure coagulation and haemostasis. The field was rinsed with water, the working sleeve was pulled out, the incision was sutured without drainage tube, and then the site was covered with a sterile dressing.

### Postoperative treatment

After the operation, nutritional nerve drugs were given. The next day, the patients wearing a waist circumference were encouraged to get out of bed and advised to avoid excessive weight-bearing, bending and twisting within one month and to moderately perform lumbar and back muscle function exercises. All patients were regularly re-examined with postoperative X-rays, 3D CT reconstruction and MRI.

### Follow‑up and data collection

**Fig. 1:**
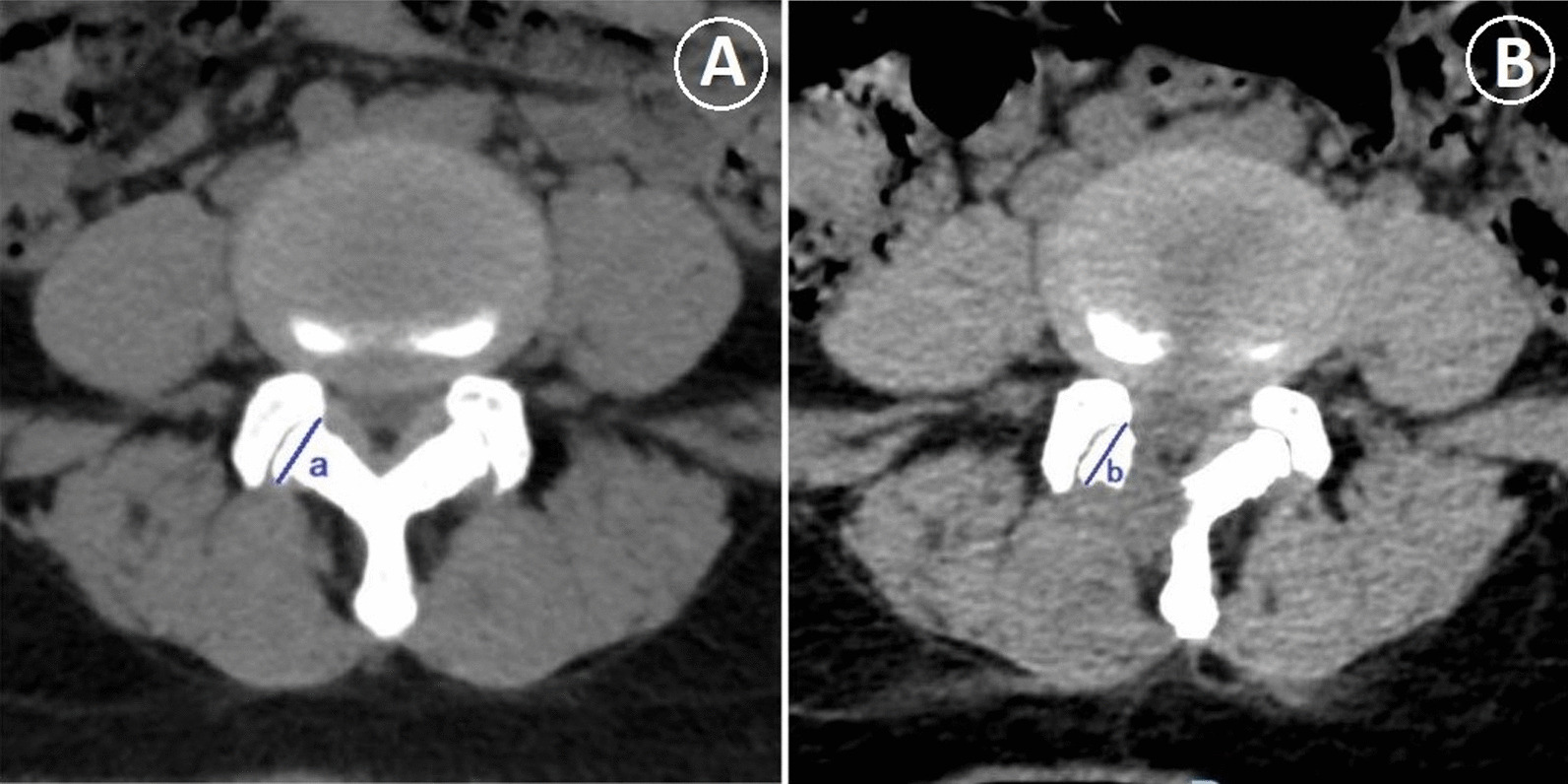
**A**, **B** The postoperative facet preservation rate was assessed by measuring the axial articular surface length of the preoperative and postoperative responsible segments on lumbar horizontal CT scan image. Postoperative facet preservation rate = b/a × 100%

The hospitalization time, operation duration, intraoperative blood loss, number of fluoroscopy exposures, incision length and number of excellent–good cases were recorded. The 3D CT axial articular surface length of the preoperative and postoperative responsible segments was measured to estimate and compare the postoperative facet preservation rate [11] (Fig. 1).

The visual analogue score (VAS) of lower back pain and leg pain was recorded before the surgery and at the third day, three months and the last follow-up after surgery. The Oswestry Disability Index (ODI) was adopted before the surgery, three months and at the last follow-up after surgery. The Macnab criteria was used to evaluate the clinical outcomes of the two groups during the last follow-up.

### Statistical analysis

All statistical analyses were performed using SPSS statistical software (version 24.0; SPSS, Inc., Chicago, IL, USA). The Shapiro‒Wilk test was used to test measurement data, and values are expressed as means ± SD or median (P25–P75). The comparison between the two groups was made by independent sample* t* test or nonparametric test, and the comparison of VAS and ODI at different time points was analysed by two-way repeated measures ANOVA. The *χ*^*2*^ test was used to test counting data. *P* < 0.05 was accepted as statistically significant.

## Results

### Baseline characteristics

A total of 230 patients (132 men and 98 women) were included in this study. The high-grade migration group included 82 patients, and the low-grade migration group included 148 patients. Patient characteristics are presented in Table 1. There was no significant difference in age, sex, body mass index (BMI), preoperative lower back and leg VAS, or preoperative ODI between the two groups (*P* > 0.05). In the high-grade migration group, one of the patients had surgery level at L2–3; nine patients, at L3–4; 40 patients, at L4–5; and 32 patients, at L5-S1. In the low-grade migration group, one of the patients had surgery level at L2–3; eight patients, at L3–4; 90 patients, at L4–5; and 49 patients, at L5-S1, with no statistical significance between the two groups (*P* > 0.05). All patients were followed up for more than 18 months after surgery, with no statistical significance between the two groups (*P* > 0.05). (Table 1).

**Table 1:** Demographic data of the two groups

Groups	High-grade migration group (*n* = 82)	Low-grade migration group (*n* = 148)	*P*-value
Age, years	49.9 ± 12.8	49.7 ± 12.1	0.913
Sex, male/Female	54:28	78:70	0.053
Body mass index, kg/l^2^	24.7 ± 2.4	24.7 ± 2.6	0.972
Disc herniation level			0.180
L2–3	1	1	
L3–4	9	8	
L4–5	40	90	
L5-S1	32	49	
Spondylolisthesis, Non/I°	50:32	106:42	0.098
VAS scores			
For lower back pain	6.6 ± 1.3	6.6 ± 1.2	0.883
For leg pain	7.0 ± 0.8	6.9 ± 1.0	0.391
ODI	70.6 ± 6.6	70.7 ± 5.3	0.918
Follow-up time, months	21(20–23)	21(20–23)	0.987

Data are expressed as the median (P25–P75), number or mean ± SD as stated

*VAS* visual analogue scale, *ODI* Oswestry Disability Index

*P* < 0.05, statistical significance

### General results of surgery

There were no significant differences in hospitalization time, operation duration, intraoperative blood loss, number of fluoroscopy exposures, incision length or postoperative facet preservation rate between the two groups (*P* > 0.05). (Table 2).

**Table 2:** Surgical outcome data

Groups	High-grade migration group (*n* = 82)	Low-grade migration group (*n* = 148)	*P*-value
Hospitalization time, days	6 (5–7)	6 (5–6)	0.594
Operative duration, min	69.0 ± 17.0	65.7 ± 17.9	0.173
Blood loss, ml	47.7 ± 11.3	44.6 ± 12.7	0.063
Number of fluoroscopy exposures	3 (2–3)	2 (2–3)	0.819
Incision length, mm	1.5 ± 0.2	1.5 ± 0.3	0.994
Facet preservation rate, %	96.7 ± 5.3	97.7 ± 4.8	0.155
Number of excellent–good cases	71	135	0.368

Data are expressed as the median (P25–P75), mean ± SD or number as stated

*P* < 0.05, statistical significance

### Clinical and functional outcomes

**Fig. 2:**
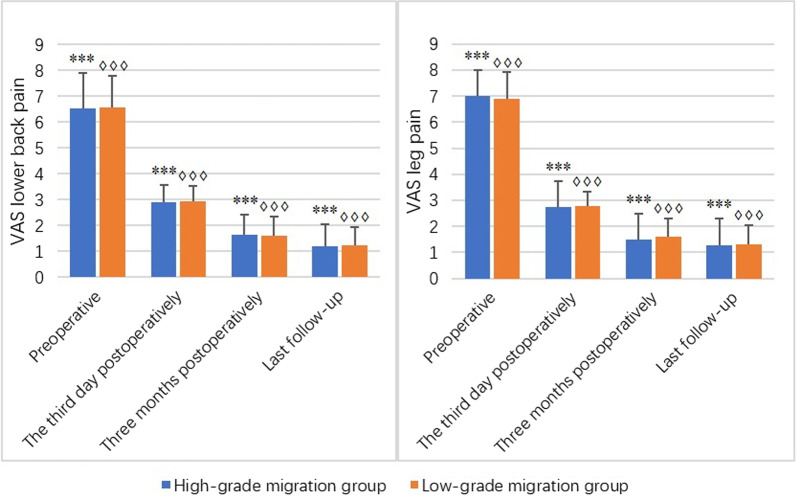
Bar charts show the results of preoperative and postoperative the third day, three months and the last follow-up VAS of lower back and leg pain in terms of mean, with the vertical line representing the SD. ***Represents the significant difference for the pairwise comparison in the high-grade migration group (*P* < 0.0001). ♢♢♢Represents the significant difference for the pairwise comparison in the low-grade migration group (*P* < 0.0001)

**Fig. 3:**
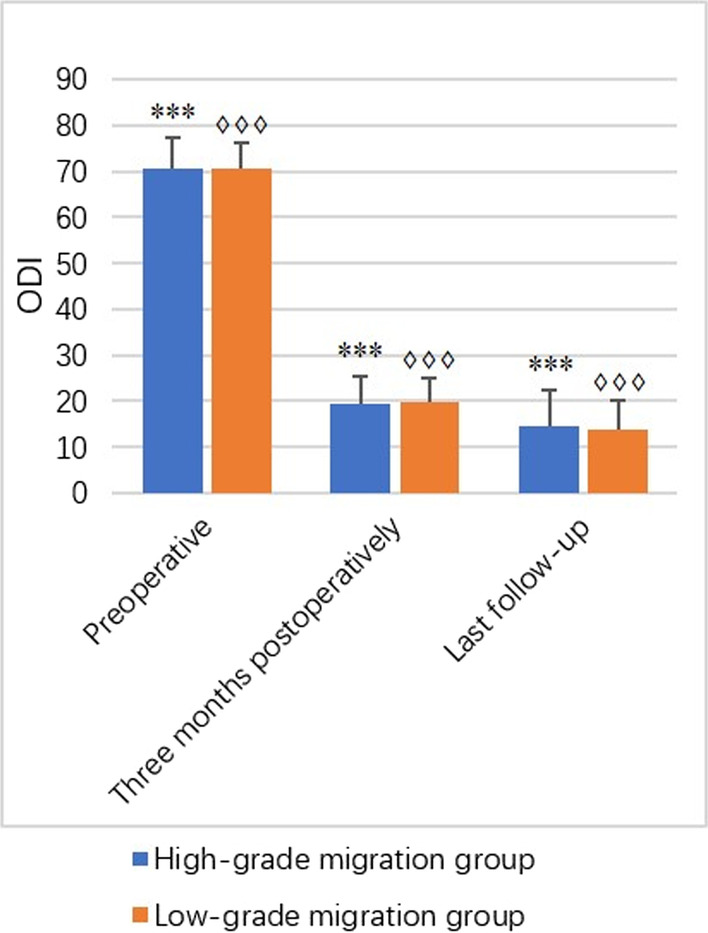
Bar charts show the results of preoperative and postoperative three months and the last follow-up ODI in terms of mean, with the vertical line representing the SD. ***Represents the significant difference for the pairwise comparison in the high-grade migration group (*P* < 0.0001). ♢♢♢Represents the significant difference for the pairwise comparison in the low-grade migration group (*P* < 0.0001)

The lower back and leg VAS and ODI were statistically significant before and after surgery in either of the two groups (*P* **< **0.001), and the comparison between the two was statistically significant in different periods in either of the two groups (*P* < 0.05). There was no significant difference in the lower back and leg VAS and ODI between the two groups in the same period (*P* > 0.05). (Fig. 2 and Fig. 3).

**Fig. 4:**
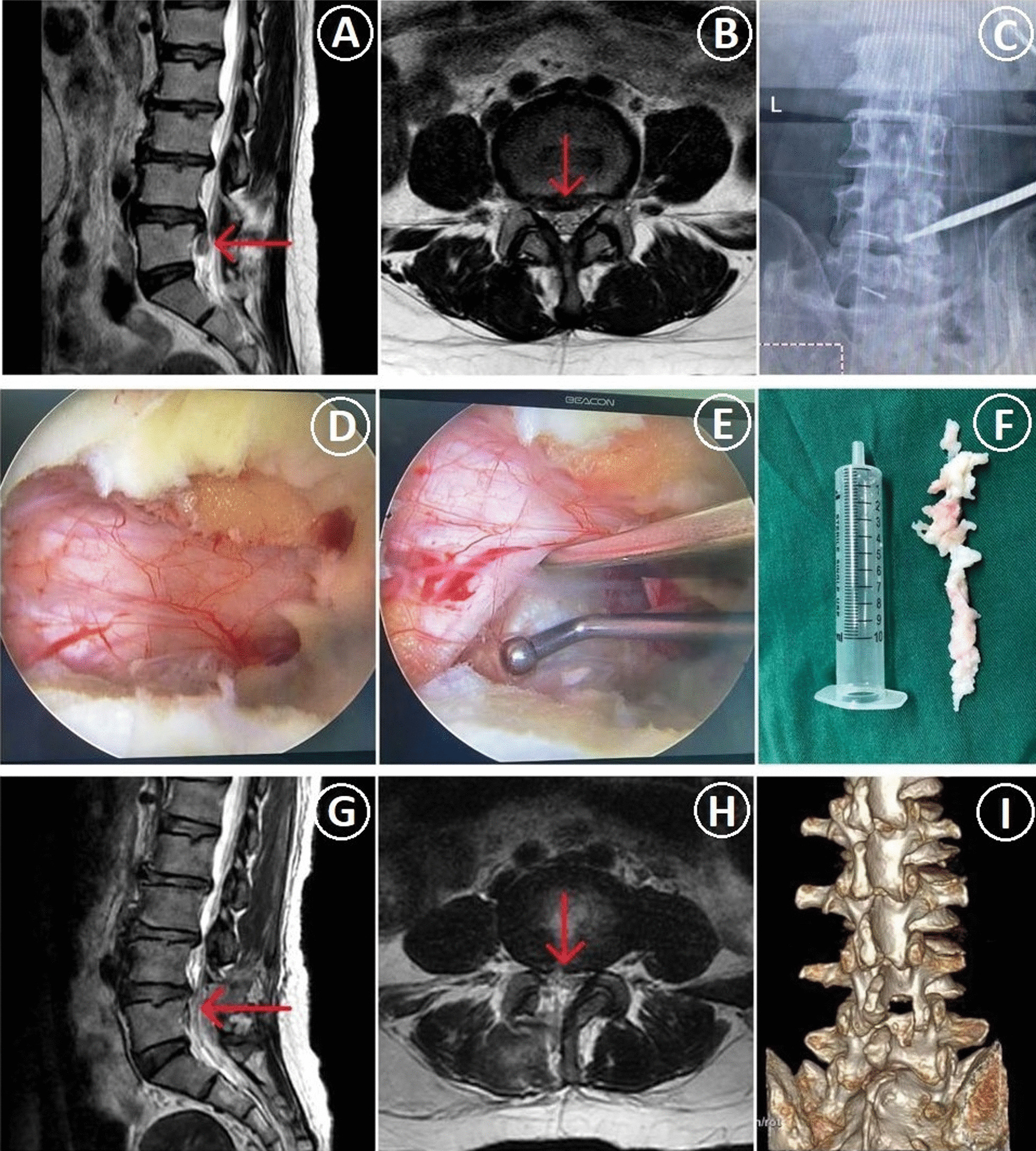
A 48-year-old female was diagnosed preoperatively with high-grade migrated LDH at the L4–5 level and underwent discectomy by OSE. **A**, **B** The preoperative T2-weighted MR images showed that the migrated intervertebral disc was located in zone 4. **C** Intraoperative anteroposterior fluoroscopy was used to confirm the responsible segment. **D**, **E** Images under the endoscope. **F** The nucleus pulposus tissue. **G**, **H** The postoperative T2-weighted MR images showed that the migrated intervertebral disc was completely removed. **I** The 3D CT showed that partial bone defected

There was no conversion to open operation during the operation. There were two cases of postoperative nerve root stimulation symptoms in the high-grade migration group and three cases in the low-grade migration group, and these symptoms disappeared after these patients received nutritional therapy about two weeks. In the high-grade migration group, one patient was reoperated by OSE technique due to recurrence at five days after surgery and postoperative effect was good at last follow-up time. The postoperative incisions of the two groups healed in one stage without complications such as infection. There was no significant difference in number of excellent–good cases between the two groups (*P* > 0.05). The overall excellent–good rate was 89.6%. Postoperative lumbar MRI examination showed that there was no residual nucleus pulposus in the spinal canal. (Fig. 4).

## Discussion

LDH is one of the most common diseases of the spine, most of which can be alleviated by conservative treatment. Migrated LDH is a more serious type, which means that the nucleus pulposus tissue breaks through the annulus fibrosus and the posterior longitudinal ligament and is free in the spinal canal and epidural space [12]. Generally, migrated LDH is often associated with nerve root function injury or abnormality. Conservative treatment has a poor effect, and surgical treatment is usually required [13]. According to the literature statistics, the high-grade migration type accounts for approximately 33% of patients with migrated LDH [2, 14]. When removing the free nucleus pulposus tissue with severe prolapse by traditional surgical methods, a large amount of soft tissue needs to be removed, and even part of the lamina and facet joints need to be removed, which causes great damage to the muscle, bone and ligament structure, results in slow recovery and easily leads to iatrogenic instability [15]. Among the current surgical methods, minimally invasive surgery is preferred over open surgery for both young and old individuals. Percutaneous endoscopic discectomy (PELD) has become a mainstream operation for LDH due to its advantages of less trauma, quick postoperative recovery and a comparable decompression effect to that of traditional surgery. However, migrated nucleus pulposus tissue, especially in high-grade migrated LDH, is easy to break when pulled because of asynechia, some fragments cannot be completely removed, and extensive exploration and complete removal of the free nucleus pulposus is often required to relieve symptoms [16, 17]. PELD still has difficulty completely removing the free nucleus pulposus separated into multiple parts due to anatomical obstruction, restriction of a single rigid duct, narrow surgical field and other factors [16, 18]. The failure rate of PELD in high-grade migrants was reported to be as high as 15.7% [19]. In recent years, some surgeons have treated migrated LDH with different modified PELD methods and have reported satisfactory results [17, 20, 21]. However, some scholars worry that the bony manipulation of the intervertebral foramina may increase the risk of postoperative lumbar instability [22, 23]. In 2013, Soliman et al. applied the UBE technique to LDH patients, and the rate of excellent and good postoperative outcomes reached 95%, making the procedure popular [24]. The UBE technique has a flexible operation and significant clinical effect in the treatment of migrated LDH, and relevant literature has also proven its clinical efficacy [9, 25].

The OSE technique is a new surgical method proposed recently. Similar to the UBE technique, it is divided into a working channel and an observation channel. The difference is that the two channels are operated in the same incision. In anaesthesia surgery, a long operative duration and large intraoperative blood loss are related factors leading to delayed resuscitation [17], and delayed resuscitation aggravates surgery-related risks. In this study, both groups had short operative duration and less blood loss, which soft tissue and bone bleeding is stopped in time, avoiding the problems mentioned above. The patients in both groups were in stable condition after surgery and had no discomfort symptoms such as headache and nausea. Traditional surgery has a long incision and large paraspinal muscle stripping area, which easily causes chronic low back pain and may lead to lumbar failure syndrome [26]. In this study, the incision length of the two groups was short, and they could go to the ground the day after surgery with less trauma. For older patients, the incidence of postoperative complications was also reduced. Too much radiation can cause varying degrees of damage to both patients and surgeons, and studies have shown that repeated exposure to radiation increases the risk of cataract [27, 28]. In this study, the number of fluoroscopy exposures in both groups were low, the location was simple and rapid, and the radiation exposure of patients and surgeons was significantly reduced. The surgical principle of maintaining the stability of lumbar vertebrae is now accepted by an increasing number of spinal surgeons. Raynor et al. concluded through animal experimental studies that more than 50% of bilateral facet resections would lead to instability in a single spinal motor segment [29]. Therefore, the removal of more than 50% of facet joints during spinal decompression would cause iatrogenic instability, and bone fusion should be performed. The biomechanical study of Teo et al. also showed that 50% fractional joint excision has a significant impact on both translational and rotational stability of movable segments [30]. None of the included cases in this study had lumbar instability, and the postoperative facet preservation rate in both groups was significantly higher than 50%, indicating that the OSE technique has a little damage to the normal bone structure of the spine and prevent the decline in lumbar strength and iatrogenic instability caused by injury to the articular process. LEE et al. treated 38 cases of high-grade migrated LDH with PELD, and the rate of excellent and good outcomes was only 78.9%. The failure rate of patients with high-grade migration (21.1%) was significantly higher than that of patients with low-grade migration, and therefore, open surgical treatment was recommended^4^. Although previous studies have shown the feasibility of PELD in the treatment of migrated LDH, the failure rate of PELD in the treatment of high-grade migrated LDH is still high (5%–22%) [19, 31, 32]. In this study, the lower back and leg VAS scores of all patients at different periods after surgery were significantly improved compared with those before, and the lower back and leg VAS and ODI scores showed no significant difference between the two groups, with a comprehensive excellent and good outcome rate of 89.6%, indicating that the OSE technique achieved good clinical efficacy in the treatment of high-grade and low-grade migrated LDH by a minimally invasive method.

Residual nucleus pulposus is one of the most important complications. Migrated LDH, especially the high-grade type, generally requires extensive exploration and complete removal of the nucleus pulposus to completely relieve the symptoms. According to the literature review, there are approximately 5%–13% of patients whose nucleus pulposus is not removed completely and who need to be operated on again [22, 33]. Choi et al. analysed 10,228 LDH patients who underwent percutaneous transforaminal endoscopic discectomy. Of the 283 cases of postoperative residual disc tissue, 70 cases were identified as migrated LDH (24.7%), and 11 cases were identified as distant high-grade migrated LDH (3.9%)^10^. In this study, postoperative MRI of all the patients showed complete removal without obvious residue, which confirmed that the OSE technique can expose and explore the structure of the spinal canal in all directions to completely remove the prolapsed free nucleus pulposus tissue. In the high-grade migration group, one patient's symptoms suddenly worsen when she exercised on the ground on the fifth day after surgery. We consider that the residual nucleus pulposus of the disc was re-extruded due to the patient’s improper activity. This patient was reoperated by OSE technique and the intraoperative evidence was relapse, and postoperative effect was available at the follow-up points. We should pay attention to the perioperative education and strengthen it for patients. The fibrous annulus can be wrinkled and shaped under the endoscopic view, which can reduce the postoperative recurrence rate. There were two cases of postoperative nerve root irritation symptoms in the high-grade migration group and three cases in the low-grade migration group, and these symptoms disappeared after symptomatic treatment with neurotrophic drugs about two weeks, which may have been caused by the large compression of the nerve root or its adhesion to the nerve root during the removal of nucleus pulposus tissue. There were no postoperative cases of dural injury. We consider the visual field of the OSE technique to be clearer than that of conventional open surgery and the enlargement of the ligamentum flavum and dural space after saline perfusion makes its separation safer and reduces the risk of dural injury. Patients without postoperative infection may benefit from continuous perfusion and irrigation during the operation. Kim et al. also considered this reason [34].

### Surgical experiences

The OSE technique has the following technical advantages: (i) The two separate operations can rotate and swing freely. Compared with PELD, there is no coaxial restriction, and the operation is flexible; (ii) Locating the lamina is simple and rapid, reducing the damage caused by radiation; (iii) The direction of the operation channel is consistent with that of the observation channel, which reduces the visual error and blind area and is conducive to complete deco; (iv) The imaging display is the same as the traditional conventional operation, and the learning requirement of arthroscopic triangulation technology [23] is lower. The learning curve of beginners is relatively smooth; (v) Microresection of the articular process reduces iatrogenic lumbar instability caused by treatment of the articular process; (vi) Continuous saline irrigation has fewer residual inflammatory mediators and reduces the incidence of postoperative pain and infection. When performing OSE surgery, attention should be given to the following: (i) There is reasonable planning of the operation procedure before surgery and careful reading of the film to determine the free degree and direction; (ii) Bleeding is stopped in time, soft tissue bleeding is stopped by electrocoagulation of appropriate power, and bone wax is used to stop bleeding; (iii) There is omnidirectional exploration of the migrated zone, especially the high-grade type, to prevent incomplete decompression or residual nucleus pulposus tissue; (iv) The rupture of fibrous annulus is treated by wrinkling and shaping it under endoscope to reduce recurrence; (v) The flow velocity is kept below 150 mL/min or the perfusion pressure at 25–30 mmHg [35, 36] during saline irrigation to reduce the risk of postoperative headache or epilepsy.

This study had several limitations. Firstly, the follow-up time of this study was short, and the sample size was small. Secondly, the study adopted a retrospective design. The long-term effects on the stability of the lumbar spine and the long-term efficacy and complications of this operation need to be further verified by large-scale prospective randomized controlled studies.

## Conclusion

The OSE technique for the treatment of migrated LDH has less trauma, quick postoperative recovery, complete removal of the nucleus pulposus and satisfactory early clinical efficacy.

## Data Availability

The datasets generated during and/or analysed during the current study are available from the corresponding author on reasonable request.

## Abbreviations

LDH: Lumbar disc herniation
OSE: One-hole split endoscope
VAS: Visual analogue score
ODI: Oswestry Disability Index
PELD: Percutaneous endoscopic lumbar discectomy
UBE: Unilateral biportal endoscope
